# Correlation between significant asymptomatic carotid artery stenosis and severity of peripheral arterial occlusive disease in the lower limb: a retrospective study on 200 patients

**DOI:** 10.1186/s12883-019-1485-1

**Published:** 2019-10-28

**Authors:** Zhongjie Pan, Ruitao Wang, Li Li, Hua Zhang

**Affiliations:** Vascular Department, Tianjin Union Medical Center, No.190, Jieyuan Road, Hongqiao District, Tianjin, 300121 China

**Keywords:** Arteriosclerosis, Carotid stenosis, Stroke

## Abstract

**Background:**

The aim of this study was to evaluate the correlation between significant asymptomatic carotid artery stenosis (ACAS) and severity of peripheral arterial occlusive disease (PAOD) in the lower limb, and to investigate the risk factors for significant ACAS in patients with lower limb PAOD.

**Methods:**

Two hundred patients with lower limb PAOD were retrospectively reviewed. Baseline data, medical history and potential risk factors were collected. Lower limb PAOD was classified as stage IIA, stage IIB, stage III and stage IV. The carotid artery stenosis was classified as significant ACAS and non-significant ACAS. Multiple logistic regression estimated odds ratio of the risk factors.

**Results:**

Compared to patients with non-significant ACAS, the patients with significant ACAS were significantly older in age and had greater percentage of cigarette-smoking andalcohol beverage consumption, and higher levels of total cholesterol and fibrinogen. There was no significant difference in sex, diabetes, hypertension, coronary heart disease and triglyceride between the two groups. The prevalence rate of significant ACAS increased with the stage of lower limb PAOD and with age. Advanced age and hypercholesteremia were risk factors for significant ACAS in this cohort. The prevalence rate of stroke increased with ACAS stage.

**Conclusion:**

The results suggested that the prevalence rate of significant ACAS was positively correlated with the severity of lower limb PAOD and age. Advanced age and hypercholesteremia appeared to be potential risk factors for significant ACAS in patients with lower limb PAOD.

## Background

Arteriosclerosis is a systemic vascular disease with carotid artery and lower limb artery as predilection sites. Asymptomatic carotid artery stenosis (ACAS) refers to presence of internal carotid/carotid bifurcation stenotic or occlusive lesions in patients without signs or symptoms of cerebrovascular disease [1]. ACAS is commonly seen in clinical practice and its diagnosis rate is increasing with assistance of modern imaging technique [2]. The incidence rate of peripheral arterial occlusive disease (PAOD) is reported to be 10% in people above 65 years old in European, and 15–20% in the population above 70 years old [3]. With an aging population and a changing dietary habit, incidence of peripheral arterial occlusive disease (PAOD) is increasing in China [4]. The incidence rate of lower limb PAOD is around 15.91% in people above 60 years old [5]. Carotid artery stenosis and lower limb PAOD usually share the same pathological changes and can co-exist. Serious ACAS and complete occlusion of carotid artery could result in fatal cerebrovascular events. It is meaningful to investigate the prevalence of significant ACAS and its risk factors in patients with lower limb PAOD. Thus, it may help for optimal screening and managing significant ACAS in clinical practice.

There are many reports on the correlation between general PAOD and ACAS and a few studies [1, 6–8] focusing on the population with lower limb PAOD. It would be clinically meaningful to obtain a better knowledge of the risk factors for significant ACAS in patients with lower limb PAOD. Therefore, this study retrospectively reviewed the patients with peripheral arterial occlusive disease in lower extremities, in order to evaluate the correlation between the prevalence of significant ACAS and the severity of lower limb PAOD. We further investigated the risk factors for significant ACAS in patients with lower limb PAOD.

## Patients and methods

### Patients

This study was approved by the ethic committee of our hospital. The patients with peripheral arterial occlusive disease in lower extremities who were admitted to our hospital between October 2013 and October 2015 were retrospectively reviewed. The inclusion criteria were patients with symptoms of intermittent claudication, rest pain and/or gangrene; peripheral arterial occlusive disease confirmed by duplex sonography, magnetic resonance angiography or digital subtraction angiography during the admission; patients with carotid artery stenosis confirmed by color doppler imaging during the admission. The exclusion criteria were patients with ischemic stroke history, patients with a history of carotid artery disease, patients allergic to contrast agent, patients with combined heart, liver and/or kidney disease, patients with artery stenosis caused by non-arteriosclerosis, and patients with blood coagulation disorder. For diagnosis of lower limb PAOD, all patients underwent duplex sonography, magnetic resonance angiography or digital subtraction angiography. Color doppler imaging was performed to diagnose ACAS and evaluate the plaque characteristics. For the asymptomatic carotid stenosis patients with mild or moderate stenosis, they were firstly managed medically with drug including high-intensity statin, antithrombotic agents and blood pressure control, and lifestyle changes including smoking cessation and low-fat diet.

### Data collection

Detailed baseline data and medical history were retrospectively collected from the medical records, including sex, age and potential risk factors [9] including coronal heart disease, hypertension, diabetes, hyperlipidaemia, hyperfibrinogenemia, cigarette-smoking and alcohol-beverage drinking history. Risk factors were defined as the following. Coronary heart disease was defined as patients with a relevant medical history and who are receiving relevant/appropriate treatment for this. Hypertension was defined as systolic pressure > 140 mmHg and diastolic pressure > 90 mmHg. The diabetes was defined as plasma glucose level ≥ 7.0 mmol/l (126 mg/dl); plasma glucose ≥11.1 mmol/l (200 mg/dl) 2 h after a 75 g oral glucose load as in a glucose tolerance test; symptoms of high blood sugar and casual plasma glucose ≥11.1 mmol/l (200 mg/dl); glycated hemoglobin (HbA1C) ≥48 mmol/mol. The hyperlipidemia was defined as the total cholesterol > 6.2 mmol/L after 12-14 h fasting. Hyperfibrinogenemia was defined as fibrinogen > 4.0 g/L. Cigarette-smoking was defined as a history of having at least one cigarette a day. Alcohol-beverage drinking was defined as a history of having at least 50 ml alcohol beverage a day.

Lower limb PAOD was classified into stage I-IV according to Fontaine R [10]. Stage I: asymptomatic and incomplete blood vessel obstruction. Stage II: mild claudication pain in limb. Stage IIA: claudication when walking a distance of greater than 200 m. Stage IIB: claudication when walking a distance of less than 200 m. Stage III: rest pain, mostly in the feet. Stage IV: necrosis and/or gangrene of the limb. The patients in stage IIB, III and IV were treated with arterial angioplasty or artificial artery transplantation; the patients in stage IIA were treated with drugs rather than surgery.

The carotid artery stenosis was classified according to Mannheim intima-media thickness consensus [11]. It was considered as arteriosclerotic plaque if the thickness between the media-adventitia interface and the intima-lumen interface was above 1.5 mm. Plaques were further classified as soft plaque, hard plaque, flat plaque and ulcerated plaque, with the first two considered as stable and the last two as unstable plaque according to previous reports [11, 12]. The severity of carotid artery stenosis was classified as mild (stenosis ≤50%) with peak systolic velocities (PSV) =125 cm/s and PSV_ICA_/ PSV_CCA_ ratio < 2.0, moderate (50% < stenosis < 70%) with PSV between 125 and 230 cm/s and PSV_ICA_/ PSV_CCA_ ratio between 2.0 and 4.0, serious (70% ≤ stenosis < 99%) with PSV > 230 cm/s and PSV_ICA_/ PSV_CCA_ ratio > 4.0, and complete occlusion [13]. In this study, the patients with stenosis ≥70% were classified as significant ACAS and patients with stenosis < 70% as non-significant ACAS.

### Statistical analysis

Statistical analysis was performed using SPSS 19.0 (SPSS Inc., Chicago, IL, USA). Data were presented as mean ± SD for continuous variables, and frequencies with percentages for categorical variables. Pearson χ^2^ test was used for categorical variables in the two cohorts and independent t-test for continuous variables. A binary logistic regression further estimated the odds ratio of the risk factors. *P* value < 0.05 indicated statistically significant.

## Results

Two hundred patients (131 males and 69 females) met the enrollment criteria and were reviewed. According to Fontaine R’s classification [10] for lower limb PAOD, there were 80 cases of stage IIA, 64 cases of stage IIB, 33 cases of stage III and 23 cases of stage IV. As for carotid artery stenosis, there were 94 cases of mild ACAS (47.0%; 68 males and 26 females), 59 cases of moderate ACAS (29.5%; 37 males and 27 females), 44 cases of serious (22.0%; 24 males and 20 females) and 3 cases of complete occlusion (1.5%; 2 males and 1 female).

Compared with patients with non-significant ACAS, the patients with significant ACAS were significantly older in age, and had greater percentage of cigarette-smoking and alcohol-beverage consumption, and higher level of total cholesterol and fibrinogen (*P* < 0.05; Table 1). There was no significant difference in sex, diabetes, hypertension, coronary heart disease and triglyceride between the two groups (*P* > 0.05).

**Table 1:** Comparison of potential risk factors between patients with significant ACAS and those with non-significant ACAS

Factors	Non-significant ACAS (153 cases)	Significant ACAS (47 cases)	*P*-value
Age (years)	58.5 ± 8.5	72.9 ± 9.1	0.032*
Sex (male)	105 (68.6%)	26 (55.3%)	0.093
Cigarette-smoking	120 (78.7%)	46 (97.9%)	< 0.001*
Diabetes	142 (92.8%)	46 (97.9%)	0.294
Hypertension	135 (88.2%)	45 (95.7%)	0.133
Coronary heart disease	148 (96.7%)	18 (38.3%)	0.707
Alcohol-beverage drinking	127 (83.0%)	45 (95.7%)	< 0.001*
Triglyceride (mmol/L)	1.33 ± 0.55	1.37 ± 0.53	0.618
Total cholesterol (mmol/L)	4.63 ± 0.96	5.02 ± 1.12	0.025*
Fibrinogen (g/L)	3.82 ± 0.91	4.18 ± 1.05	0.038*

*ACAS* Asymptomatic carotid artery stenosis

*Significant *P* value

The prevalence of significant ACAS increased with the stage of lower limb PAOD (χ^2^ = 28.2, *P* = 3.17 × 10^− 6^; Table 2). It also increased with age (χ^2^ = 7.78, *P* = 0.020; Table 3). Multi-variate logistic regression showed advanced age and hypercholesteremia were independent risk factors for significant ACAS in patients with lower limb PAOD (Table 4).

**Table 2:** The incidence rate of significant ACAS increased with the stage of lower limb PAOD

Lower limb PAOD	ACAS (%)^a^		Total number	*P*-value^b^	Odds ratios (95% confidence interval)
	Non-significant	Significant			
Stage IIA	72 (90.0%)	8 (10.0%)	80	< 0.001*	0.362 (0.188–0.695)
Stage IIB	52 (81.3%)	12 (18.8%)	64	0.277	0.751 (0.440–1.284)
Stage III	18 (54.6%)	15 (45.5%)	33	0.001*	2.713 (1.485–4.954)
Stage IV	11 (47.8%)	12 (52.2%)	23	0.001*	3.551 (1.677–7.518)
Total number	153	47	200		

*ACAS* Asymptomatic carotid artery stenosis, *PAOD* Peripheral arterial occlusive disease

*Significant *P*-value

^a^χ^2^ = 28.2, *P* = 3.17 × 10^−6^; Spearman’s rho = 0.352, *P* = 3.13 × 10^−7^

^b^After Bonferroni correction α’ = 0.013

**Table 3:** The incidence rate of significant ACAS increased with the age of the patients

Age	ACAS (%)^a^		Total number	*P*-value^b^	Odds ratios (95% confidence interval)
	Non-significant	Significant			
50–59	38 (90.5%)	4 (9.5%)	42	0.016*	0.343 (0.129–0.910)
60–69	62 (77.5%)	18 (22.5%)	80	0.785	0.945 (0.627–1.425)
≥ 70	53 (68.0%)	25 (32.1%)	78	0.023*	1.536 (1.087–2.169)
Total number	153	47	200		

*ACAS* Asymptomatic carotid artery stenosis

*Significant *P*-value

^a^χ^2^ = 7.78, *P* = 0.020; Spearman’s rho = 0.194, *P* = 0.006

^b^After Bonferroni correction α’ = 0.017

**Table 4:** Multi-variate logistic regression of the risk factors for significant asymptomatic carotid artery stenosis

Factors	β	OR (95% CI)	*P*-value
Age	0.076	1.079 (1.055–1.104)	0.042*
Sex	0.694	1.352 (0.933–1.959)	0.114
Cigarette-smoking	0.353	1.325 (0.926–1.872)	0.182
Alcohol-beverage drinking	0.345	1.415 (0.913–1.866)	0.126
Hypertension	0.852	2.423 (1.225–4.449)	0.063
Diabetes	0.488	1.565 (0.954–1.568)	0.081
Coronary heart disease	0.455	1.576 (0.977–1.732)	0.667
Hypercholesteremia	0.546	2.013 (1.076–3.325)	0.033*
Hyperlipidemia	−0.077	0.964 (0.832–1.213)	0.972

*Significant *P*-value

## Discussion

In the present study, we found that the prevalence rate of significant ACAS increased with the stage of lower limb PAOD. It also increased with age. Multi-variate logistic regression showed advanced age and hypercholesteremia were independent risk factors for significant ACAS. In the patients above 70 years old, the percentage of significant ACAS is 32.10%, which was significantly higher (*P* < 0.05) than those below 70 years old. Among the patients with lower limb PAOD under stage IV, there were 12 cases of significant ACAS (12/23, 52.2%) and it was higher than patients in other stages. But Mirsharifi R’s research [14] pointed out that carotid artery stenosis had correlation with diabetes, hypertension, dyslipidemia, coronary heart disease and ABI, while it had nothing to do with age. Pilcher J et al. [15] studied 200 cases of PAOD and reported that there was no correlation between the degree of carotid artery stenosis and lower limb arteriosclerosis. Marek et al’s [16] study showed the carotid artery stenosis ≥50% had close correlation with age > 65 years old, ABI < 0.7 and carotid bruit. Mirsharifi et al. [14] reported that carotid artery stenosis was correlated with diabetes, hypertension, hyperlipidemia, coronary heart disease, severity of presenting symptom and ABI, and it had significant association with age. Song et al. [8] also evaluated the prevalence of asymptomatic carotid artery stenosis in patients with lower extremities PAOD from Peking Union Medical College Hospital. They found that the prevalence of asymptomatic carotid artery stenosis was higher in patients with lower extremities PAOD, which was in accordance with our results. Smoking, coronary artery disease and hypertension were related risk factors in their study while in our study there was no significant difference in hypertension and coronary heart disease between the two groups (*P* > 0.05). We thought that this difference might be due to the characteristics of the study population and the sample size.

In this study, the percentage of significant ACAS increased with the severity of lower limb PAOD (*P* < 0.05), which was in accordance with previous report [17]. Therefore, screening for significant ACAS in lower limb PAOD is important, especially in those patients with risk factors. In this regard, it may benefit the patients with more severe PAOD to get screened regularly for ACAS. Thus, proper preventive measures could be taken in time to even reduce morbidity or stroke prevalence. It is meaningful in clinical screening especially when the prevalence of PAOD is increasing in China [4].

## Conclusions

There are some limitations in this study, such as small sample size and single center. It is necessary to perform a further study with a larger sample in multiple centers. Besides, the generalizability of the study results is limited because the study participants are only Chinese people in North China. In conclusion, the prevalence of significant ACAS increased with the severity of lower PAOD and age. Advanced age and hypercholesteremia were potential risk factors for significant ACAS in patients with lower limb PAOD. We suggest that patients with lower limb PAOD and the potential risk factors may benefit from the screening for significant ACAS.

## Data Availability

The data and materials are available if required.

## Abbreviations

ACAS: Asymptomatic carotid artery stenosis
PAOD: Peripheral arterial occlusive disease
